# No evidence that women using oral contraceptives have weaker preferences for masculine characteristics in men’s faces

**DOI:** 10.1371/journal.pone.0210162

**Published:** 2019-01-10

**Authors:** Urszula M. Marcinkowska, Amanda C. Hahn, Anthony C. Little, Lisa M. DeBruine, Benedict C. Jones

**Affiliations:** 1 Faculty of Public Health, Department of Health Sciences, Medical College Jagiellonian University, Cracow, Poland; 2 Department of Psychology, Humboldt State University, Humboldt, Arcata, California, United States of America; 3 Department of Psychology, University of Bath, Bath, United Kingdom; 4 Institute of Neuroscience and Psychology, University of Glasgow, Glasgow, United Kingdom; Iwate Medical University, JAPAN

## Abstract

Previous research has suggested that women using oral contraceptives show weaker preferences for masculine men than do women not using oral contraceptives. Such research would be consistent with the hypothesis that steroid hormones influence women’s preferences for masculine men. Recent large-scale longitudinal studies, however, have found limited evidence linking steroid hormones to masculinity preferences. Given the relatively small samples used in previous studies investigating putative associations between masculinity preferences and oral contraceptive use, we compared the facial masculinity preferences of women using oral contraceptives and women not using oral contraceptives in a large online sample of 6482 heterosexual women. We found no evidence that women using oral contraceptives had weaker preferences for male facial masculinity than did women not using oral contraceptives. These findings add to a growing literature suggesting that links between reproductive hormones and preferences are more limited than previously proposed.

## Introduction

Masculine facial characteristics in men are hypothesized to be cues of a strong, heritable immune system but also of reduced willingness to invest in relationships [1–3]. Given a proposed trade off between the benefits and costs of choosing a masculine mate, women could maximize the potential genetic and investment benefits of their mate choices by mating with men with masculine facial characteristic when fertile, while forming long-term relationships with men with relatively feminine facial characteristics [1–3].

Consistent with this proposal, many studies have reported stronger preferences for masculine male faces when women were tested during the ovulatory (i.e., high-fertility) phase of their menstrual cycle [3, 4]. However, these studies have been criticized for being underpowered and relying on self-report data to estimate women’s position in the menstrual cycle [5, 6]. Recent longitudinal studies that directly addressed these criticisms by testing large samples of women and assessing women’s hormonal status using measured hormone levels have found no compelling evidence that women’s preferences for masculine male faces track changes in their hormonal status or fertility [7, 8].

Research examining possible associations between oral contraceptive use and women’s masculinity preferences is a second line of evidence that steroid hormones influence preferences for masculine men. The rationale for these studies is that oral contraceptive use will cause weaker masculinity preferences because it is negatively correlated with fertility [8–12]. Some of these studies have reported that women using oral contraceptives showed weaker preferences for masculine male faces than did women not using oral contraceptives [9, 10]. Additionally, one longitudinal study has reported that women’s preferences for masculine male faces decreased after they started using oral contraceptives [11]. However, other recent studies found that women using oral contraceptives reported *stronger* preferences for masculine male faces than did women not using oral contraceptives [8, 12] and that changing use of oral contraceptives did not have a significant effect on women’s preferences for masculinity in men’s faces [8].

To further examine associations between oral contraceptive use and masculinity preferences, we carried out a large-scale study to compare preferences for masculine characteristics in men’s faces in a sample of women who reported using oral contraceptives (N = 1857) and a sample of women not using oral contraceptives (N = 4625). If oral contraceptive use is linked to weaker masculinity preferences, women using oral contraceptives would be expected to show stronger preferences for feminized versions of men’s faces than women not using hormonal contraceptives.

Some previous studies have suggested that women’s masculinity preferences might be related to their age [13] and partnership status [3] and that hormonal status might also influence women’s judgments of same-sex faces [14]. The rationale for these predictions is that older women may have stronger preferences for masculine men because they look older [13], that partnered women have stronger preferences for masculine men because such women already have an investing partner (i.e., a partner who will invest resources, such as time, in the relationship [3]). Steroid hormones might also alter women’s perceptions of feminine women by altering the extent to which women are motivated to affiliate with same-sex individuals who appear to be caring social partners, altering the extent to which women monitor high-quality competitors for mates, or simply as a low-cost functionless by-product of adaptations for assessing potential mates[14]. Consequently, we also collected and analyzed data on women’s age, partnership status, and preferences for masculinity-femininity in women’s faces.

## Materials and methods

### Participants

Six thousand four hundred and eighty-two heterosexual women (mean age = 23.01 years, SD = 5.34 years; age range 16 to 40 years) took part in this online study, which was run at faceresearch.org. One thousand eight hundred and fifty-seven of the women reported using the oral contraceptive pill and 4625 of the women reported using no form of hormonal contraceptive. No participants included in our analyses reported being pregnant or using any other form of hormonal supplement. An additional 402 women who reported using forms of hormonal contraceptives other than the oral contraceptive pill or hormonal supplements were tested, but were excluded from the data set prior to analyses. One thousand eight hundred and thirty-three of the women reported being in a romantic relationship at the time of testing and 1703 of the women reported that they were not (the remaining 2946 women did not report their partnership status, for more information see S1 Supplementary Materials). All participants provided informed consent and all aspects of the study were approved by the University of Glasgow’s Psychology Ethics Committee (OCMATE project).

### Stimuli

**Fig 1:**
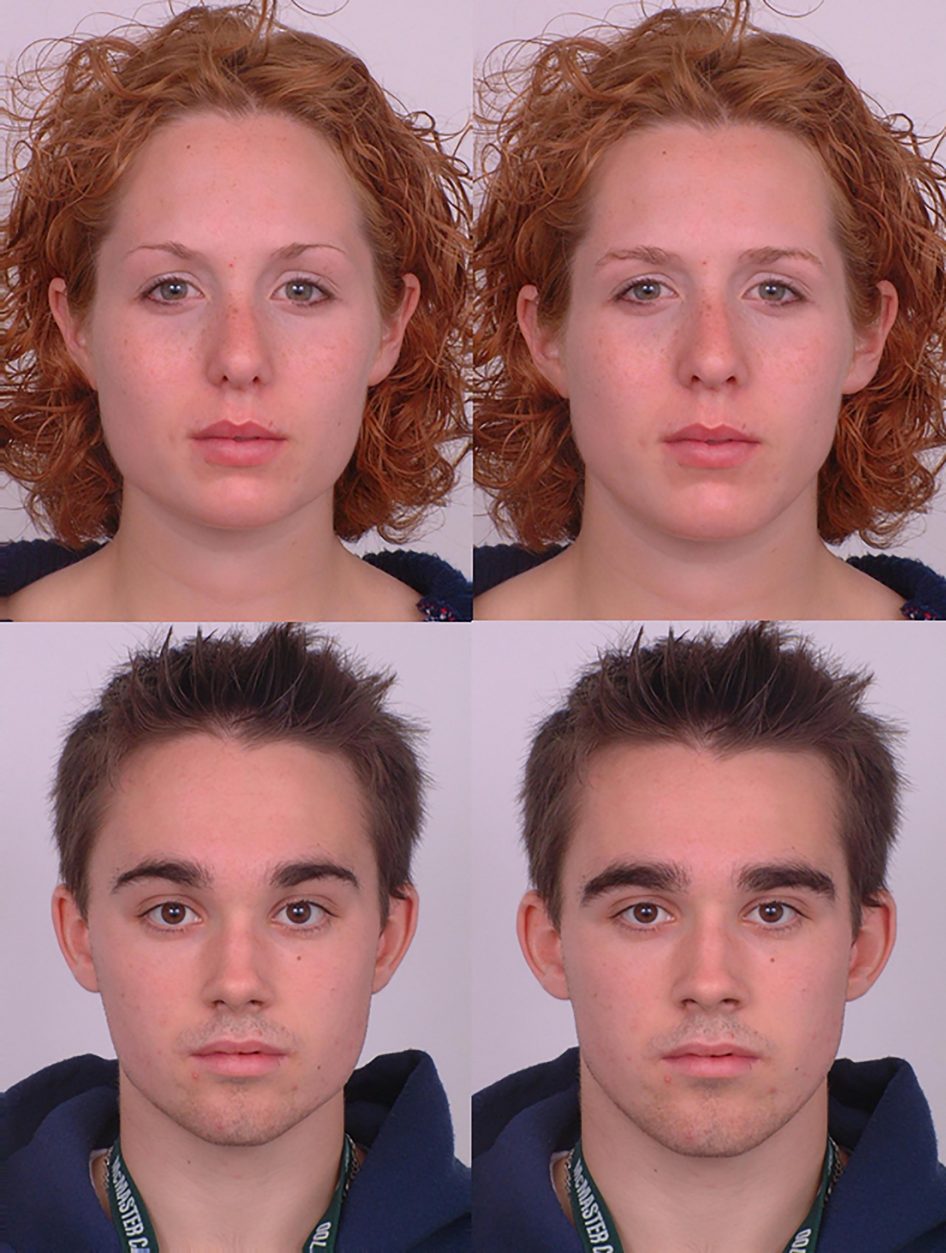
Examples of masculinized (right) and feminized (left) versions of male and female face images used in the study.

Following previous studies of individual differences in women’s preferences for masculine faces [7–10], we used prototype-based image transformations to objectively manipulate sexual dimorphism of 2D shape in face images. First, male and female prototype (i.e. average) faces were manufactured using established computer graphic methods that have been widely used in studies of face perception [15]. These prototypes were manufactured using face images of 20 young White male adults and 20 young White female adults, respectively. Next, 50% of the linear differences in 2D shape between symmetrized versions of the male and female prototypes were added to or subtracted from face images of 20 young White male adults and 20 young White female adults. This process creates masculinized and feminized versions of the individual face images that differ in sexual dimorphism of 2D shape and that are matched in other regards. Examples of masculinized and feminized versions of male and female faces are shown in Fig 1. These stimuli are publicly available [16]. The individuals in this manuscript have given written informed consent (as outlined in PLOS consent form) to publish these case details.

### Procedure

Participants were shown the 40 pairs of face images and were asked to choose the face in each pair that was more attractive. Participants also indicated the strength of these preferences by choosing from the options ‘slightly more attractive’, ‘somewhat more attractive, ‘more attractive’, and ‘much more attractive’. The order in which pairs of faces were shown was fully randomized and the side of the screen on which any particular image was shown was also fully randomized. Responses were coded using a 0 (masculinized face judged much more attractive than feminized face) to 7 (feminized face judged much more attractive than masculinized face). These preference scores were centered on chance before being used in our analyses.

## Results

Analyses were conducted using R v3.4.3. Data, analysis code, and full results are publicly available at https://osf.io/dcz5t/ and in S1 Supplementary Materials.

First, we analyzed preference scores using a mixed effect model (using lmer [17], and lmerTest [18]) with the within-subject factor *sex of face* (effect coded male = 0.5, female = -0.5), the between-subject factor *use of oral contraceptives* (effect coded yes = 0.5, no = -0.5), and the covariate *participant age* (z-scored). Random intercepts were included for participant and stimulus, with random slopes specified maximally (following recommendations in [19, 20]).

There was a significant main effect of *sex of face* (estimate = -1.023, se = 0.140, t = -7.333, p < .001), whereby women showed stronger femininity preferences when judging women’s than men’s faces. There was a significant interaction between *sex of face* and *use of oral contraceptives* (estimate = -0.060, se = 0.026, t = -2.319, p = .021). There were no other significant effects (both p > .11).

The interaction between sex of face and use of oral contraceptives was explored by repeating the analysis above separately for male and female faces. Women using oral contraceptives showed stronger femininity preferences than women not using oral contraceptives when assessing women’s faces (estimate = 0.056, se = 0.017, t = 3.266, p = .001), but not when assessing men’s faces (estimate = -0.010, se = 0.022, t = -0.467, p = .641). For female faces, the positive intercept (estimate = 1.007, se = 0.083, t = 12.164, p < .001) indicated femininity preferences were significantly above chance. For male faces, the near-zero intercept (estimate = -0.017, se = 0.112, t = -0.155, p = 0.879) indicated femininity preferences were not significantly different from chance. For female faces, there was a significant positive effect of participant age (estimate = 0.019, se = 0.007, t = 2.585, p = .010), indicating that older women had stronger preferences for female femininity. For male faces, there was a significant negative effect of participant age (estimate = -0.025, se = 0.009, t = -2.732, p = .006), indicating that older women had stronger preferences for male masculinity.

Next, we repeated the analysis above, this time with *partnership status* (effect coded partnered = 0.5, unpartnered = -0.5) as an additional between-subject factor (note that the sample size is smaller for this analysis because 2946 women did not report their partnership status and, consequently, could not be included in this analysis). As in the previous analysis, there was a significant main effect of *sex of face* (estimate = -1.033, se = 0.141, t = -7.307, p < .001), indicating that women showed stronger femininity preferences when judging women’s than men’s faces. There were no other significant effects (all p > .11).

## Discussion

Our analyses revealed no evidence that women using oral contraceptives showed weaker preferences for masculine men than did women who were not using oral contraceptives. Our data do not then support the hypothesis that oral contraceptive use is associated with (and potentially causes) weakened preferences for masculinity in male faces [9–11]. The current study’s null result for oral contraceptive use and masculinity preference adds to a growing body of evidence challenging the claim that women’s preferences for exaggerated sexual dimorphism in men are related to their reproductive hormones [7, 8].

Although we found no evidence that preferences for masculine men differed between women using and not using oral contraceptives, our analysis suggested that women using oral contraceptives showed stronger preferences for feminine women. Some researchers have suggested that stronger preferences for feminine women when women are in conditions associated with low fertility could reflect reduced intrasexual competitiveness [21]. However, given this association between oral contraceptive use and femininity preferences was not apparent in our analysis of the reduced, but still large, sample (the 3536 women for who partnership data were available), we suggest it is most likely a false positive.

In line with previous studies, we found that women preferred feminized versions of women’s faces over masculinized versions [22]. However, women did not prefer masculinized versions of men’s faces over feminized versions in the current study, which is consistent with the generally mixed findings for the attractiveness of masculine male faces in the facial attractiveness literature [2].

By contrast with Little et al. [10], we found no evidence that partnered women showed stronger preferences for masculine men than unpartnered women did. This null result suggests that the positive effect of partnership status on women’s preferences for masculine men reported previously is not robust or is more complex than previously thought. Although we found that older women tended to show stronger preferences for masculine men (see also [9]), this relationship was very weak.

Like previous studies using this type of between-subjects design to test for evidence of the possible effects of oral contraceptive use on women’s masculinity preferences that reported significant between-group differences [10], we did not distinguish between (and do not have data on) the types and brands of oral contraceptives women were using. Thus, it is possible that comparisons taking into account these factors could yet reveal differences in the masculinity preferences of women not using oral contraceptives and women using specific types and/or brands (see, e.g., [23] for a study reporting correlations between oral contraceptive composition and aspects of women’s mating psychology). While we do not discount this possibility, it would be at odds with null results reported in recent high-powered studies testing for links between endogenous steroid hormones and masculinity preferences [7, 8].

Similarly, we did not consider possible effects of participant ethnicity on face preferences. Since participant ethnicity may be correlated with oral contraceptive use, it would be useful to control for participant ethnicity in future research using this type design.

In conclusion, we replicated the finding that women show stronger preferences for feminine shape characteristics in women’s faces than they do in men’s faces [8, 21, 22]. However, we found no evidence that oral contraceptive users showed weaker preferences for masculine men than do women not using oral contraceptives. These findings add to a growing body of evidence suggesting that oral contraceptive use has limited association with women’s mate preferences [8, 12] and mating psychology [24].

## Supporting information

S1 Supplementary Materials All supporting information has been included in the “S1 Supplementary Materials”. (PDF)
