# Supplementary material for: No evidence that women using oral contraceptives have weaker preferences for masculine characteristics in men’s faces
Source: PLoS One. 2019 Jan 10;14(1):e0210162. doi: 10.1371/journal.pone.0210162 (PMC6328097; doi:10.1371/journal.pone.0210162)
Supplement: S1 Supplementary Materials — (PDF) [file pone.0210162.s001.pdf]

Code

No evidence that women using oral contraceptives have weaker preferences for masculine characteristics in men's faces

Ben Jones

Hide

```
library(lmerTest)

library(tidyverse)

sessionInfo()
```

```
R version 3.4.3 (2017-11-30)

Platform: x86_64-apple-darwin15.6.0 (64-bit)

Running under: macOS High Sierra 10.13.3



Matrix products: default

BLAS: /System/Library/Frameworks/Accelerate.framework/Versions/A/Frameworks/vec
Lib.framework/Versions/A/libBLAS.dylib

LAPACK: /Library/Frameworks/R.framework/Versions/3.4/Resources/lib/libRlapack.d
ylib



locale:

[1] en_GB.UTF-8/en_GB.UTF-8/en_GB.UTF-8/C/en_GB.UTF-8/en_GB.UTF-8



attached base packages:

[1] stats     graphics  grDevices utils     datasets  methods   base



other attached packages:

 [1] lmerTest_2.0-33 lme4_1.1-14     Matrix_1.2-12   bindrcpp_0.2

 [5] forcats_0.3.0   stringr_1.3.0   dplyr_0.7.4     purrr_0.2.4

 [9] readr_1.1.1     tidyr_0.8.0     tibble_1.4.2    ggplot2_2.2.1

[13] tidyverse_1.2.1



loaded via a namespace (and not attached):

 [1] Rcpp_0.12.15        lubridate_1.7.3     lattice_0.20-35

 [4] assertthat_0.2.0    rprojroot_1.2       digest_0.6.13

 [7] psych_1.7.8         R6_2.2.2            cellranger_1.1.0

[10] plyr_1.8.4          backports_1.1.1     acepack_1.4.1

[13] evaluate_0.10.1     httr_1.3.1          pillar_1.1.0
```

```
[16] rlang_0.2.0         lazyeval_0.2.1      readxl_1.0.0
[19] data.table_1.10.4-3 rstudioapi_0.7      minqa_1.2.4
[22] nloptr_1.0.4        rpart_4.1-11        checkmate_1.8.5
[25] rmarkdown_1.8       labeling_0.3        splines_3.4.3
[28] foreign_0.8-69      htmlwidgets_0.9     munsell_0.4.3
[31] broom_0.4.3         compiler_3.4.3      modelr_0.1.1
[34] pkgconfig_2.0.1     base64enc_0.1-3     mnormt_1.5-5
[37] htmltools_0.3.6     nnet_7.3-12         tidyselect_0.2.3
[40] htmlTable_1.9       gridExtra_2.3       Hmisc_4.0-3
[43] crayon_1.3.4        MASS_7.3-47         grid_3.4.3
[46] nlme_3.1-131        jsonlite_1.5        gtable_0.2.0
[49] magrittr_1.5        scales_0.5.0        cli_1.0.0
[52] stringi_1.1.6       reshape2_1.4.2      latticeExtra_0.6-28
[55] xml2_1.2.0          Formula_1.2-2       RColorBrewer_1.1-2
[58] tools_3.4.3         glue_1.2.0          hms_0.4.1
[61] parallel_3.4.3      survival_2.41-3     yaml_2.1.16
[64] colorspace_1.3-2    cluster_2.0.6       rvest_0.3.2
[67] knitr_1.19          bindr_0.1           haven_1.1.1
```

Hide

```
#http://faceresearch.org/res/data/myqueries?id=193

data_raw <- read_csv("pill_40_faces.csv")
```

```
Parsed with column specification:
cols(
  .default = col_integer(),
  user_id = col_character(),
  sex = col_character(),
  age = col_double(),
  date = col_date(format = ""),
  ethnicity = col_character(),
  pill = col_character()
)
See spec(...) for full column specifications.
```

Demographics

Hide

```
data_raw %>%
  summarise(
    n = n(),
    mean_age = mean(age, na.rm = T),
    age_sd = sd(age, na.rm = T)
  )
```

n <int>	mean_age <dbl>	age_sd <dbl>
6482	23.00946	5.341338

1 row

pill <chr>	partner <int>	n <int>	mean_age <dbl>	age_sd <dbl>
none	0	1349	21.55953	4.837185
none	1	1171	24.58617	5.929258

none	NA	2105	23.49392	5.743972
ocp	0	354	21.51243	3.924228
ocp	1	662	22.64577	4.358747
ocp	NA	841	22.84364	4.586664

6 rows

Hide

```
count(data_raw)
```

n <int>
6482

1 row

Hide

```
count(data_raw, pill)
```

pill <chr>	n <int>
none	4625
ocp	1857

2 rows

Hide

```
count(data_raw, partner)
```

partner <int>	n <int>
0	1703
1	1833
NA	2946

3 rows

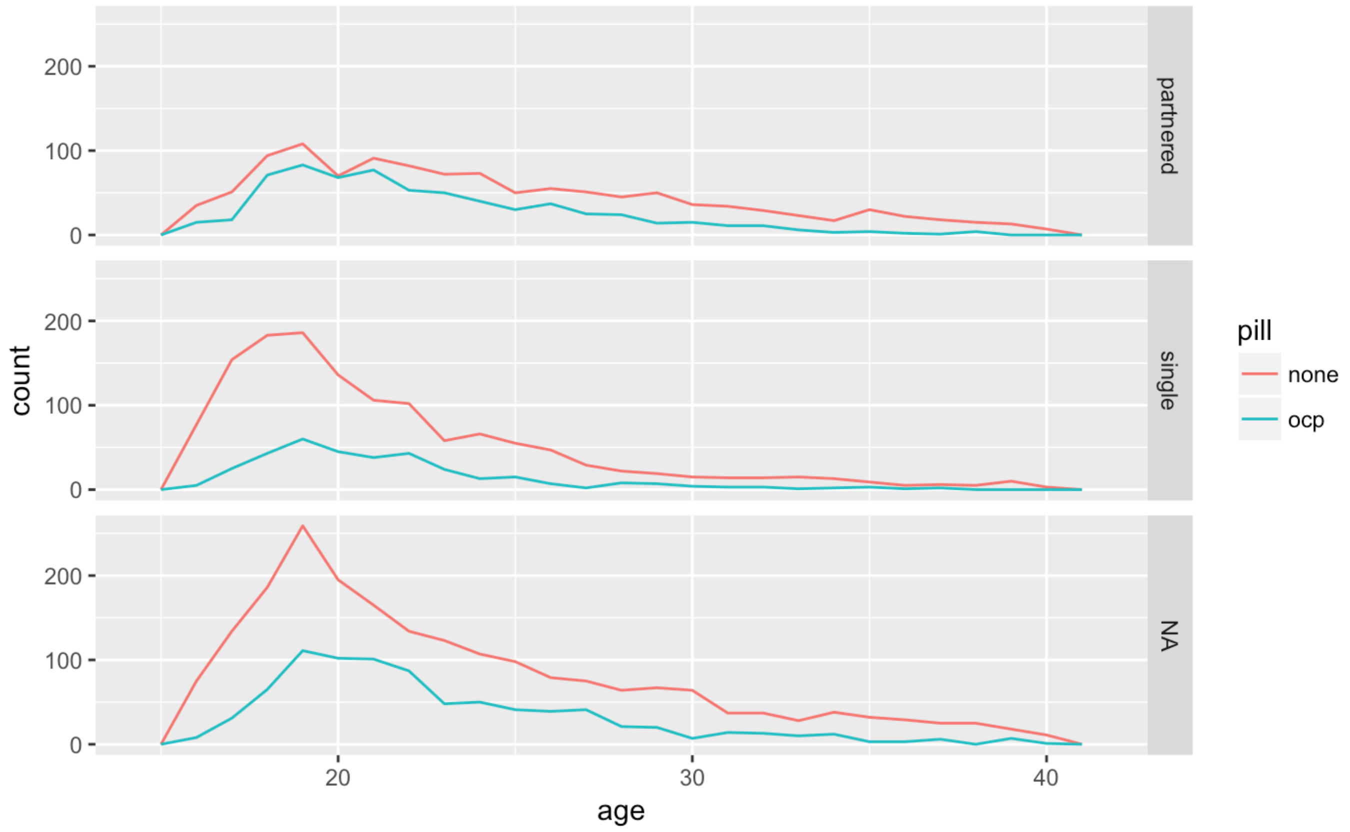

Data processing

Hide

```
data_long %>%
  group_by(user_id, pill, stim_sex) %>%
  summarise(rating = mean(rating.c)) %>%
  group_by(stim_sex) %>%
  summarise(
    mean_rating = mean(rating),
    sd_rating = sd(rating)
  )
```

stim_sex <chr>	mean_rating <dbl>	sd_rating <dbl>
female	0.9950633	0.5996646
male	-0.0152345	0.7251992

2 rows

Hide

```
data_long %>%
  group_by(user_id, pill, stim_sex) %>%
  summarise(rating = mean(rating.c)) %>%
  group_by(stim_sex, pill) %>%
  summarise(
    mean_rating = mean(rating),
    sd_rating = sd(rating)
  )
```

**stim_sex** <chr>	**pill** <chr>	**mean_rating** <dbl>	**sd_rating** <dbl>
female	none	0.97976216	0.6094405
female	ocp	1.03317178	0.5729803
male	none	-0.01326486	0.7363924
male	ocp	-0.02014001	0.6967102

4 rows

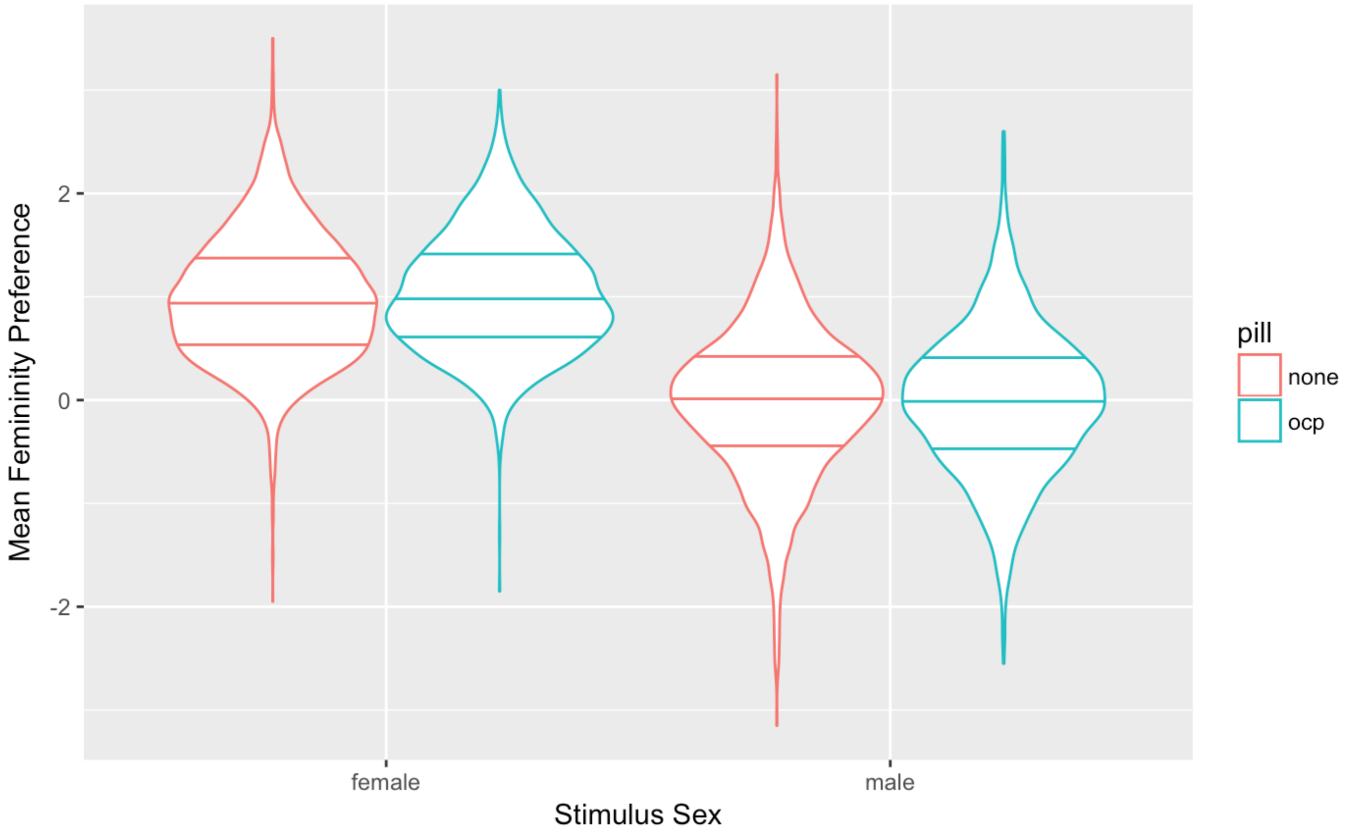

Analyses with Pill

Effect	Estimate	Std. Error	df	t value	Pr(>|t|)
<chr>	<dbl>	<dbl>	<dbl>	<dbl>	<dbl>
(Intercept)	0.495	0.070	38.730	7.085	0.000
stim_sex.e	-1.023	0.140	38.575	-7.333	0.000
pill.e	0.023	0.014	680.454	1.587	0.113
age.s	-0.003	0.006	6479.005	-0.434	0.664
stim_sex.e:pill.e	-0.060	0.026	460.767	-2.319	0.021

5 rows

Male faces only

Effect	Estimate	Std. Error	df	t value	Pr(>|t|)
<chr>	<dbl>	<dbl>	<dbl>	<dbl>	<dbl>
(Intercept)	-0.017	0.112	19.278	-0.155	0.879
pill.e	-0.010	0.022	261.192	-0.467	0.641
age.s	-0.025	0.009	6479.055	-2.732	0.006

3 rows

Female faces only

Effect	Estimate	Std. Error	df	t value	Pr(>|t|)
<chr>	<dbl>	<dbl>	<dbl>	<dbl>	<dbl>
(Intercept)	1.007	0.083	19.321	12.164	0.000
pill.e	0.056	0.017	358.667	3.266	0.001
age.s	0.019	0.007	6479.028	2.585	0.010

3 rows

Analyses with Partnership

Effect	Estimate	Std. Error	df	t value	Pr(>|t|)
<chr>	<dbl>	<dbl>	<dbl>	<dbl>	<dbl>
(Intercept)	0.488	0.071	39.154	6.899	0.000
stim_sex.e	-1.033	0.141	38.904	-7.307	0.000
pill.e	0.029	0.019	1066.588	1.556	0.120
age.s	0.002	0.009	3532.047	0.251	0.802
partner.e	0.026	0.017	3532.047	1.503	0.133

stim_sex.e:pill.e	-0.041	0.033	761.503	-1.226	0.220

6 rows
